# Effervescent-Assisted Dissolving Microneedle Array Patches for Localized Tetracycline Delivery: A Three-Layer Design for Rapid-Onset Antimicrobial Therapy in Superficial Skin Infections

**DOI:** 10.1021/acsomega.5c13276

**Published:** 2026-02-02

**Authors:** Sonthaya Chaiarwut, Chasuda Choipang, Pairayaphak Ngamplang, Pitt Supaphol

**Affiliations:** † The Petroleum and Petrochemical College, 26683 Chulalongkorn University, Bangkok 10330, Thailand; ‡ Research Unit on Herbal Extract-Infused Advanced Wound Dressing, Chulalongkorn University, Bangkok 10330, Thailand

## Abstract

Dissolving microneedle array patches (DMNAPs) offer promise for minimally invasive antimicrobial therapy but face challenges in achieving rapid drug release and complete needle deposition. This study developed and characterized a novel three-layer DMNAP incorporating an effervescent separation mechanism for enhanced tetracycline hydrochloride (TCH) delivery to infected superficial tissues. DMNAPs were fabricated using sequential casting with drug-loaded poly­(vinyl alcohol) (PVA) microneedles, an effervescent separation layer (sodium bicarbonate/tartaric acid), and a polyvinylpyrrolidone (PVP) backing. Mechanical properties, skin penetration efficiency, drug release kinetics, antimicrobial efficacy against *Escherichia coli* ATCC 25922 and *Staphylococcus aureus* ATCC 25923, and biocompatibility using human dermal fibroblasts were systematically evaluated. The optimized PVA-1.0/PVP0.75 formulation achieved insertion forces of 0.848 ± 0.054 N/needle with penetration depths of 426.1 ± 16.8 μm and 96% penetration efficiency in ex vivo porcine skin. The effervescent mechanism enabled complete needle detachment within 60 s through CO_2_-mediated separation. TCH release followed Korsmeyer–Peppas kinetics (*R* ^2^ > 0.93, *n* = 0.43–0.45), achieving >90% cumulative release within 24 h through quasi-Fickian diffusion. The system demonstrated potent bactericidal activity with >99.99% reduction of both bacterial strains within 6 h at concentrations exceeding 4 × MIC. Qualitative preliminary ex vivo antimicrobial assessment on infected porcine skin confirmed complete suppression of *E. coli* and substantial inhibition of *S. aureus* growth. Human dermal fibroblast viability remained >82% for therapeutic formulations (0.5–1.0 mg/mL TCH), confirming biocompatibility. This effervescent-assisted DMNAP platform addresses critical limitations of current microneedle technologies, offering painless, self-administrable antimicrobial therapy with minimized systemic exposure for treating antibiotic-resistant superficial infections.

## Graphical abstract

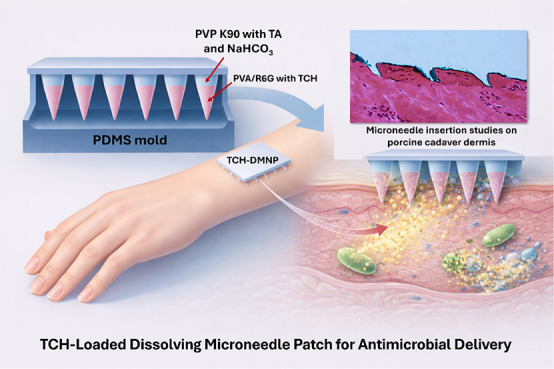

## 1 Introduction

Microneedles (MNs) have emerged as transformative tools for transdermal drug delivery, evolving from early silicon-based microelectromechanical systems (MEMS) to sophisticated polymeric platforms incorporating diverse biomaterials.^1,2^ Among various microneedle designs, dissolving microneedles (DMNs) composed of biocompatible, water-soluble polymers represent a particularly promising approach for localized therapeutic delivery.^3^ These systems create temporary micropores in the skin, enabling direct delivery of therapeutic agents to viable tissue layers while circumventing first-pass hepatic metabolism, thereby enhancing bioavailability and reducing systemic side effects.^4,5^

The advantages of DMN-based delivery over conventional hypodermic injections are substantial: painless administration due to minimal nerve stimulation, elimination of sharps waste and associated biohazards, potential for self-administration improving patient compliance, and precise control over drug loading and release kinetics.^6,7^ These features position DMNs as ideal platforms for treating superficial bacterial infections, where localized, sustained antimicrobial delivery can maximize therapeutic efficacy while minimizing systemic exposure and associated adverse effects.

Despite significant advances, current DMN technologies face persistent challenges in achieving rapid and complete needle deposition in tissue, particularly for applications requiring immediate therapeutic action. Previous approaches, including sol–gel systems, polymer-coated designs, and core–shell architectures, have demonstrated controlled release capabilities but often suffer from mechanical fragility, prolonged dissolution times requiring extended patch application, complex multistep fabrication processes, and inconsistent drug loading efficiency.^8–10^ These limitations compromise both manufacturing scalability and clinical utility, particularly for wound care applications where patient comfort and ease of use are paramount.

To address these challenges, recent innovations have explored effervescent-based separation mechanisms for microneedle systems. Li et al.^11^ pioneered this approach for long-acting contraceptive delivery, demonstrating that acid–base reactions generating CO_2_ could facilitate rapid needle detachment while maintaining structural integrity during insertion. Their system employed poly­(lactic-*co*-glycolic) acid (PLGA) microneedles designed for sustained release of levonorgestrel over more than one month, with 40% drug loading in a biodegradable matrix that slowly releases the hormone as the polymer degrades.^11^ The PLGA-based design achieved MN separation within approximately 10 s in phosphate-buffered saline, with 96 ± 4% detachment efficiency and 90 ± 4% drug delivery efficiency in porcine skin ex vivo.^11^ While this approach proved highly effective for long-acting hormonal delivery requiring month-long release kinetics, its applicability to antimicrobial therapy—where rapid drug release is essential for immediate bacterial suppression—remained unexplored. Subsequently, Liu et al.^12^ applied this principle to metformin delivery in diabetic rats, confirming the versatility of effervescent separation across different therapeutic applications. However, the integration of effervescent technology with antimicrobial delivery systems remains underexplored, despite its potential to revolutionize wound care management.

The present study differentiates itself from the pioneering PLGA-based effervescent microneedle system^11^ through deliberate selection of hydrophilic polymer matrices—poly­(vinyl alcohol) (PVA) and polyvinylpyrrolidone (PVP)—specifically optimized for antimicrobial applications requiring rapid drug release. While PLGA microneedles are designed to remain embedded in tissue and slowly biodegrade over weeks to months, releasing encapsulated drugs through polymer erosion,^11^ wound infections demand immediate therapeutic intervention with high local antibiotic concentrations. The water-soluble PVA matrix employed here dissolves rapidly upon contact with tissue fluid, enabling biphasic release kinetics characterized by initial burst release (>70% within 60 min) followed by sustained delivery—a profile ideally suited for combating acute bacterial colonization while maintaining antimicrobial coverage during early wound healing. Furthermore, the fabrication methodology differs substantially: whereas PLGA microneedles require organic solvent casting (diglyme/water) and extended drying at elevated temperatures,^11^ the aqueous-based PVA system permits straightforward room-temperature processing compatible with thermosensitive antimicrobial agents. This fundamental difference in release mechanism—dissolution-mediated versus degradation-controlled—represents a critical distinction enabling the effervescent separation concept to be translated from long-acting hormonal delivery to rapid-onset antimicrobial therapy.

Tetracycline hydrochloride (TCH) is a broad-spectrum antibiotic exhibiting potent activity against both Gram-positive and Gram-negative bacteria through inhibition of bacterial protein synthesis.^13^ TCH binds to the 30S ribosomal subunit, preventing aminoacyl-tRNA attachment to the ribosomal A site and subsequent peptide chain elongation, ultimately leading to bacteriostatic or bactericidal effects depending on concentration and bacterial strain.^14^ Recent studies have demonstrated the efficacy of TCH in microneedle-based delivery systems for wound healing applications, including double-layer designs for diabetic wounds,^15,16^ silk fibroin platforms for transdermal antibiotic delivery,^17^ and immunomodulatory patches for periodontal regeneration.^18^ These investigations consistently highlight TCH’s compatibility with various polymeric matrices and sustained-release characteristics, establishing it as an ideal model antimicrobial agent for advanced transdermal delivery platforms.

While systemic TCH administration can cause gastrointestinal disturbances, photosensitivity, and potential development of resistant bacterial strains, localized delivery via DMNAPs minimizes these adverse effects by achieving high drug concentrations at the infection site while maintaining low plasma levels.^19,20^ Recent comparative studies have demonstrated that microneedle-mediated delivery of TCH achieves superior area-under-curve (AUC) values and sustained-release profiles compared to intravenous administration, despite lower peak concentrations, confirming the advantages of this delivery route.^21^

This investigation presents the development and comprehensive characterization of TCH-loaded DMNAPs incorporating a three-layer architecture with integrated effervescent separation technology. The system combines drug-loaded PVA microneedles for therapeutic delivery, an effervescent layer (sodium bicarbonate/tartaric acid) for rapid CO_2_-mediated needle detachment, and a PVP backing for structural support and handling. We systematically evaluate mechanical properties ensuring reliable skin penetration, drug release kinetics and mathematical modeling of release mechanisms, antimicrobial efficacy through both quantitative time-kill assays and qualitative preliminary ex vivo assessment on infected porcine skin, and biocompatibility using human dermal fibroblasts. While current evaluations focus on in vitro and ex vivo assessments, this work provides essential foundational data supporting future in vivo investigations and clinical translation of this platform for treating superficial bacterial infections, particularly those involving antibiotic-resistant pathogens where localized high-dose delivery may overcome resistance mechanisms.

## 2 Materials and Methods

### 2.1 Materials

Polydimethylsiloxane (PDMS) molds [13 mm × 13 mm patch size, 10 × 10 array, 1000 μm needle height (*H*), 1000 μm tip spacing (*S*), 500 μm base dimension (*B*)] were purchased from MySkinRecipes (Thailand). Polyvinylpyrrolidone K90 (PVP K90) was obtained from Tokyo Chemical Industry (Japan). Sodium bicarbonate (NaHCO_3_) and L-(+)-tartaric acid (TA) were purchased from KEMAUS (Australia). Poly­(vinyl alcohol) (PVA, synthesis grade, 115 000 g/mol) was obtained from Loba Chemie (India). Tetracycline hydrochloride (TCH) and Rhodamine 6G (R6G) were purchased from Sigma-Aldrich (USA). Ethanol (AR grade) was obtained from RCI Labscan (Thailand). Bacterial strains, Gram-positive *Staphylococcus aureus* ATCC 25923 and Gram-negative *Escherichia coli* ATCC 25922, were sourced from Thai Can Biotech (Thailand).

### 2.2 Preparation of Casting Solutions

Three distinct solutions were prepared for sequential fabrication of the multilayered DMNAP structure. For the drug-containing first layer, PVA was dissolved in deionized (DI) water at 70 °C for 4 h with continuous stirring. Following complete dissolution, R6G (1.645 g, 1 μM) was added as a fluorescent marker, followed by TCH at varying concentrations (0.5, 1.0, or 2.0 mg/mL). The effervescent second layer solution was prepared by dissolving PVP K90 in ethanol, followed by addition of 4% w/v TA and 5% w/v NaHCO_3_. The backing third layer consisted of 40% w/v PVP K90 dissolved in a DI water–ethanol mixture (30:70 v/v). Table 1 summarizes the compositions of all formulations investigated.

**Table 1:** Composition of DMNAP and TCH-DMNAP Formulations

	Layer 1^a^			Layer 2		Layer 3		
Formulation	PVA (g)	DI (mL)	Tetracycline Hydrochloride (mg/mL)	PVP K90 (g)	EtOH (mL)	PVP K90 (g)	DI water (mL)	Ethanol (mL)
PVA-0.5/PVP0.75	0.50	5.00	-	0.75	2.50	0.40	0.30	0.70
PVA-0.5/PVP1.00	0.50		-	1.00				
PVA-1.0/PVP0.75	1.00		-	0.75				
PVA-1.0/PVP1.00	1.00		-	1.00				
0.5 TCH-DMNAP	1.00		0.50	0.75				
1.0 TCH-DMNAP	1.00		1.00	0.75				
2.0 TCH-DMNAP	1.00		2.00	0.75				

a The quantity of Rhodamine 6G for layer 1 is 1.645 g.

### 2.3 Fabrication of TCH-DMNAPs

The multilayered DMNAPs were fabricated using a sequential casting method (Figure 1). Initially, the drug-containing solution was cast into PDMS molds and centrifuged at 2,500 rpm for 2 min to ensure complete filling of the conical cavities. Excess solution was removed by scraping, and the filled molds were dried in a desiccator at room temperature for 24 h. Subsequently, the effervescent layer solution was applied and centrifuged at 6,000 rpm for 6 min to achieve uniform distribution while preventing intermixing with the drug layer. After another 24-h drying period, the backing layer solution was applied without centrifugation to form the final structural support. The completed patches were demolded after complete drying and stored in sealed containers with desiccant until use.

**Figure 1:**
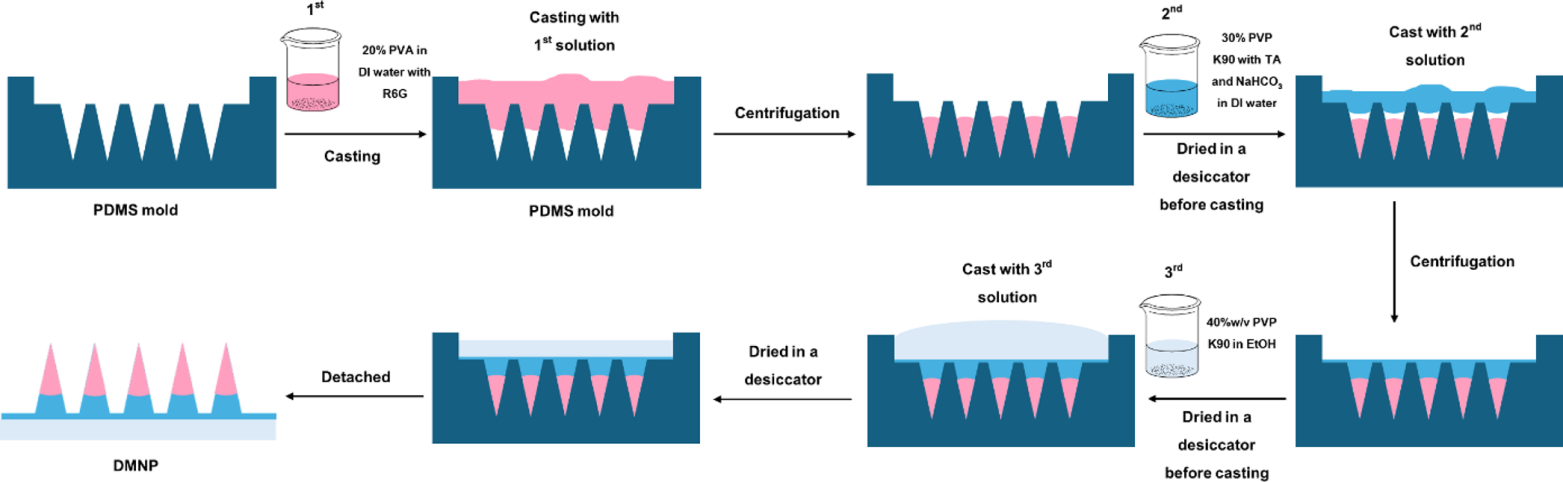
Schematic illustration of the three-layer TCH-DMNAP fabrication process using sequential casting methodology.

### 2.4 Skin Insertion Studies

Ex vivo skin penetration was evaluated using hairless porcine skin, which serves as a validated model for human skin due to similar histological structure and biomechanical properties.^22^ The skin was mounted on a rigid support plate, and DMNAPs were applied with thumb pressure for 60 s. Following patch removal, the insertion sites were stained with tissue-marking dye for 10 min. After removing excess dye, penetration efficiency was quantified by counting stained micropores using digital microscopy (Seek Inter, i-Measure HD). For histological analysis, treated skin samples were embedded in paraffin, sectioned using a microtome, and stained with hematoxylin and eosin (H&E). Cross-sectional images were acquired using a Leica RM2255 microscope to measure insertion depth and assess tissue disruption. Penetration efficiency was quantified through 10 independent patch applications (*n* = 10), with manual counting of successfully formed micropores.

### 2.5 Mechanical Characterization and Effervescence Evaluation

Mechanical properties were assessed using a Universal Testing Machine (Lloyd LRX-Plus, Lloyd Instruments, UK) equipped with a 500 N load cell. Compression tests were performed at 0.2 mm/min crosshead speed under ambient conditions. Force–displacement curves were recorded, and the force required for 0.5 mm displacement was determined as the insertion force metric. Each formulation was tested in triplicate to ensure statistical reliability. Effervescent behavior was evaluated by securing DMNAPs in glass dishes with needles oriented upward, followed by water immersion. Time-lapse imaging captured the separation dynamics, with particular attention to bubble formation and needle detachment kinetics.

### 2.6 Drug Loading and Release Studies

#### 2.6.1 Drug Content and Release Kinetics

Drug loading was quantified by completely dissolving individual TCH-DMNAPs in 10 mL phosphate-buffered saline (PBS, pH 7.4) under gentle agitation.^23^ TCH concentration was determined by UV–vis spectrophotometry at 370 nm (Evolution 300, Thermo Scientific) using a calibration curve (*R* ^2^ > 0.99). Total drug content per patch and average loading per needle were calculated based on the 10 × 10 array configuration as the ratio of the measured drug content to the mass of the microneedle patch material, according to the following equation:

1 $$\mathrm{Drug}\;\mathrm{loading}\;(\mu g/\mathrm{mg})=\frac{\mathrm{Mass}\;\mathrm{of}\;\mathrm{TCH}\;\mathrm{in}\;a\;\mathrm{patch}\left(\mu g\right)}{\mathrm{Mass}\;\mathrm{of}\;\mathrm{patch}\;\mathrm{material}\;\left(\mathrm{mg}\right)}$$

In vitro release studies were conducted by immersing TCH-DMNAPs in 10 mL PBS at 37 °C with constant agitation. At predetermined intervals (0, 5, 10, 20, 40, 60, 180, 300, 420, 720, and 1440 min), 5 mL aliquots were withdrawn and immediately replaced with fresh PBS to maintain sink conditions. TCH concentration in collected samples was measured spectrophotometrically, and cumulative release profiles were constructed.

#### 2.6.2 Release Kinetics Model-Fitting

The release data of TCH from the TCH-DMNAPs were analyzed using established mathematical models to elucidate the underlying release mechanisms.^24,25^ The models applied are presented below:

Zero-order model: where drug release is independent of concentration:

2 $$Q_{t}=Q_{0}+K_{0}t$$

First-order model: where release depends on the concentration of the remaining drug:

3 $$\ln Q_{t}=\ln Q_{0}+K_{1}t$$

Higuchi model: describing release as a diffusion process based on Fick’s law:

4 $$Q_{t}=K_{H}\sqrt{t}$$

Korsmeyer–Peppas model: used to describe release from polymeric systems when the mechanism is not well-known or involves multiple phenomena:

5 $$\frac{M_{t}}{M_{\infty }}=K_{\mathrm{KP}}t^{n}$$

where *Q*_*t*_ represents the cumulative amount released at time *t*, *Q*_0_ is the initial amount, *K*_0_ is the zero-order release rate constant, *K*_1_ is the first-order release rate constant, *K*_H_ is the Higuchi dissolution constant, $\frac{M_{t}}{M_{\infty }}$ is the fractional release, *K*_KP_ is Korsmeyer–Peppas release rate constant, and *K*_HC_ is Hixson–Crowell release rate constant. The release exponent *n* in the Korsmeyer–Peppas model indicates the release mechanism, i.e., *n* ≤ 0.43 (Fickian diffusion), 0.43 < *n* < 0.85 (anomalous transport), and *n* ≥ 0.85 (Case-II transport).

### 2.7 Antimicrobial Evaluation

#### 2.7.1 MIC and MBC Determination

Minimum inhibitory concentration (MIC) and minimum bactericidal concentration (MBC) were determined following Clinical and Laboratory Standards Institute (CLSI) protocols. MIC refers to the lowest concentration of an antimicrobial agent that visibly inhibits bacterial growth, while MBC is the minimum concentration required to kill 99.9% of the bacterial population under defined conditions. TCH solutions in Tryptic Soy Broth (TSB) were prepared at concentrations ranging from 0.01 to 10,000 μg/mL using 2-fold serial dilutions. Bacterial suspensions (*E. coli* ATCC 25922 and *S. aureus* ATCC 25923) were standardized to 0.5 McFarland turbidity (1.5 × 10^8^ CFU/mL, where CFU = colony-forming units) and diluted to 5 × 10^6^ CFU/mL in TSB. Each TCH dilution was mixed with bacterial suspension and incubated at 37 °C for 24 h. MIC was identified as the lowest concentration showing no visible growth. For MBC determination, samples from wells showing no growth were plated on TSB agar and incubated for 24 h. MBC was defined as the lowest concentration achieving a 99.9% reduction in viable bacterial cells.

#### 2.7.2 Time-Kill Kinetics

Bactericidal kinetics were evaluated using time-kill assays^26^ to assess the antimicrobial efficacy of microneedle patches. Bacterial suspensions were prepared at 5 × 10^5^ CFU/mL using a 0.5 McFarland standard and cultured in TSB. Each sample was exposed to microneedle patches and incubated at 37 °C under continuous agitation. At predetermined time points (0, 3, 6, 12, and 24 h), 20 μL aliquots were withdrawn and replaced with sterile saline to maintain volume. The collected aliquots were serially diluted, and 10 μL of each dilution was plated on sterile TSB agar. Plates were incubated overnight at 37 °C, after which CFU were counted. Bacterial viability at each time point was compared to untreated controls, and the percentage reduction in bacterial count was calculated using eq 6:

6 $$\mathrm{Bacterial}\;\mathrm{Reduction}\;(\%)=\frac{N_{\mathrm{control}}-N_{\mathrm{specimen}}}{N_{\mathrm{control}}}$$

where *N*_control_ is the number of colonies in the control (CFU/mL), and *N*_specimen_ is the number of colonies in the specimens (CFU/mL).

#### 2.7.3 Preliminary Ex Vivo Qualitative Antimicrobial Screening on Infected Porcine Skin

The qualitative antimicrobial activity of TCH-loaded dissolving microneedle array patches was evaluated using an ex vivo infected porcine skin model. Commercial pork skin with attached subcutaneous tissue was purchased frozen from a local supermarket, thawed overnight at 4 °C, and cut into approximately 3 × 3 cm^2^ pieces. The skin was rinsed with phosphate-buffered saline (PBS, pH 7.4) to remove residual blood and surface debris, then gently blotted dry. The epidermal surface was wiped with 70% (v/v) ethanol and air-dried in a biosafety cabinet, followed by 10–15 min exposure to ultraviolet light to reduce background microbial contamination while maintaining tissue structural integrity.

Bacterial suspensions of *S. aureus* ATCC 25923 and *E. coli* ATCC 25922 were prepared from overnight cultures grown in tryptic soy broth (TSB). Cultures were adjusted to 0.5 McFarland standard (∼1.5 × 10^8^ CFU/mL) and diluted with PBS to obtain a working inoculum of approximately 1 × 10^6^ CFU/mL. A 1 × 1 cm^2^ area was marked on the epidermal surface of each skin piece, and 100 μL of bacterial suspension was dispensed onto the marked area and spread evenly using a sterile pipette tip. Inoculated samples were left at room temperature in the biosafety cabinet for 15–30 min to allow bacterial adhesion, then incubated at 37 °C for 1–2 h to establish superficial infection.

Following infection, skin samples were assigned to three experimental conditions: (i) untreated infected control (no patch), (ii) neat DMNAP (drug-free microneedle patch), and (iii) TCH-loaded microneedle patch containing 2.0 mg/mL TCH in the casting solution (2.0 TCH-DMNAP). For treated groups, a single microneedle patch (10 × 10 array) was aligned to fully cover the 1 cm^2^ infected region and applied using firm thumb pressure for 60 s to ensure needle insertion. Patches were covered with occlusive film (Parafilm or transparent dressing) to maintain skin contact and prevent drying. All samples were then incubated at 37 °C for 24 h.

After 24 h incubation, the occlusive film and microneedle patches were carefully removed. The entire 1 cm^2^ area was swabbed thoroughly using a sterile cotton swab premoistened with PBS, employing horizontal, vertical, and circular strokes to maximize bacterial collection. Each swab was immediately streaked directly onto tryptic soy agar (TSA) using standard quadrant-streak technique without dilution. Plates were incubated at 37 °C for 18–24 h, after which bacterial growth was evaluated qualitatively. Visible colonies indicated bacterial survival (positive), while complete absence of visible colonies indicated bacterial suppression (negative). The 2.0 TCH-DMNAP group was compared with the untreated infected control and neat DMNAP groups to assess the antimicrobial efficacy of microneedle-delivered TCH on infected porcine skin.

### 2.8 Cytotoxicity Assessment

#### 2.8.1 Cell Culture

In accordance with ISO 10993–5:2009, in vitro cytotoxicity was assessed using the 3-(4,5-dimethylthiazol-2-yl)-2,5-diphenyltetrazolium bromide (MTT) direct contact method with human dermal fibroblasts (HDFs).^27,28^ HDFs, selected for their role in extracellular matrix production and wound healing, were cultured in complete medium containing Dulbecco’s Modified Eagle’s Medium (88%), fetal bovine serum (10%), antibiotic/antimycotic solution (1%), and l-glutamine (1%) at 37 °C in a humidified 5% CO_2_ atmosphere. Medium was refreshed every 2–3 days to expand the cell population for seeding.

#### 2.8.2 MTT Indirect Viability Assay

Cytotoxicity was evaluated using the indirect contact method in accordance with ISO 10993–5:2009 guidelines.^29,30^ Test specimens were sterilized via ultraviolet (UV) irradiation for 20–30 min and subsequently immersed in 1 mL of complete medium (CM) for 24 h to prepare extract media. After the extraction period, the media were filtered to remove any particulates prior to cell exposure. HDFs were seeded at a density of 10,000 cells per well in 96-well tissue culture polystyrene (TCPS) plates and incubated overnight at 37 °C in a humidified atmosphere containing 5% CO_2_ to allow for cell attachment. The following day, the filtered extract media were introduced into each well and incubated for an additional 24 h under the same conditions. Cell viability was assessed using the MTT assay. A solution of MTT reagent (5 mg/mL) was added to each well and incubated for 4 h, allowing viable cells to reduce the MTT to insoluble formazan crystals. After incubation, the medium was carefully removed, and dimethyl sulfoxide (DMSO) was added to solubilize the formazan. Absorbance was measured spectrophotometrically at 570 nm and cell viability was calculated as

7 $$\mathrm{Cell}\;\mathrm{Viability}\;(\%)=\frac{OD_{570,\mathrm{Sample}}}{OD_{570,\mathrm{Blank}}}\times 100$$

where OD_570, Sample_ is the mean value of the measured optical density (OD) of the 100% extracts of the test sample, and OD_570, Blank_ is the mean value of the measured optical density of the blanks.

### 2.9 Statistical Analysis

Data are presented as means ± standard deviations from triplicate experiments.^31^ Statistical analyses were performed using SPSS Statistics v25 (IBM Corporation). One-way ANOVA with least significant difference (LSD) post hoc tests determined statistical significance at *p* < 0.05.

## 3 Results and Discussion

### 3.1 Morphological Characterization and Effervescent Behavior

Digital microscopy revealed well-defined pyramidal microneedle structures with dimensions of 1000 μm height, 1000 μm tip spacing, and 500 μm base diameter (Figure 2), conforming to the PDMS mold specifications. The three-layered architecture was clearly visible in cross-sectional analysis, with the drug-containing PVA layer and effervescent PVP layer each comprising approximately 50% of the needle height. This equal distribution is strategically designed to ensure adequate drug loading while maintaining sufficient effervescent capacity for rapid separation. The uniform needle geometry across the entire 10 × 10 array confirms the reproducibility of the sequential casting fabrication method, which is critical for achieving consistent therapeutic performance.

**Figure 2:**
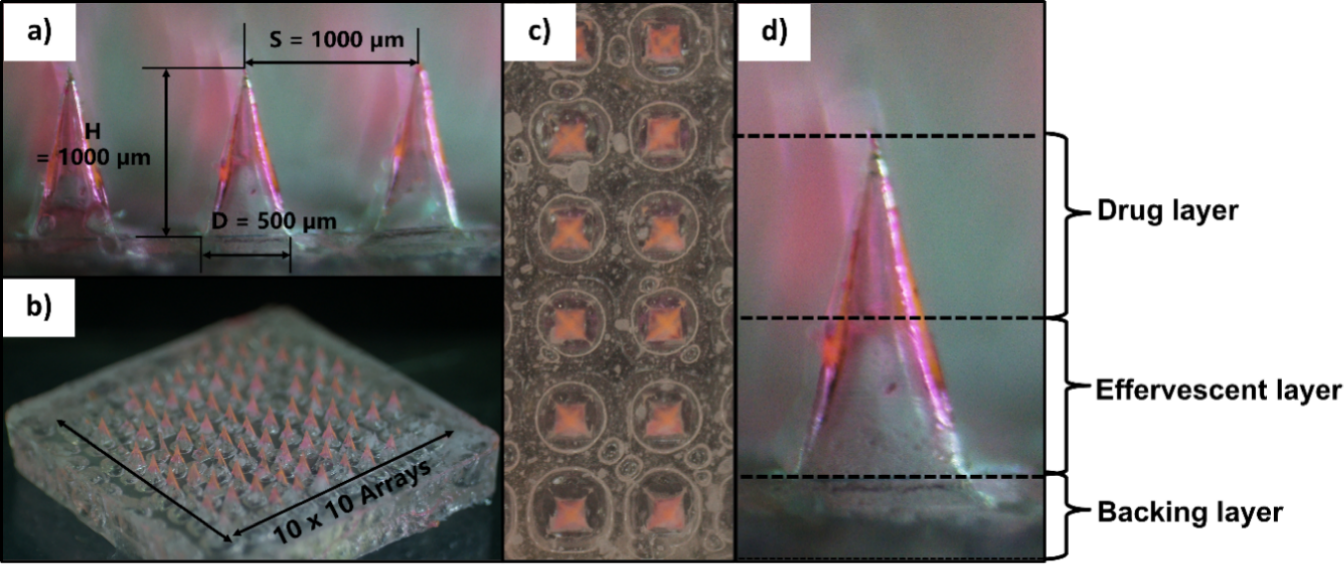
Morphological characterization of TCH-DMNAPs: (a) Side view showing individual microneedle dimensions (*H* = 1000 μm, *S* = 1000 μm, *D* = 500 μm); (b) Angled view of complete 10 × 10 array; (c) Top view demonstrating uniform needle arrangement; (d) Magnified single needle showing distinct multilayer composition.

The effervescence studies demonstrated rapid and controlled needle separation upon moisture exposure (Figure 3). Within 15 s of water contact, initial bubble formation was observed at the interface between the effervescent and backing layers, driven by the acid–base reaction between sodium bicarbonate and tartaric acid. By 60 s, most needles had successfully detached from the backing, driven by CO_2_ generation according to the following reaction:

8 $${\mathrm{NaHCO}}_{3}{\;+\;C}_{4}H_{6}O_{6}\;(\mathrm{aq})\to {\mathrm{NaC}}_{4}H_{5}O_{6}{\;+\;H}_{2}{O+\mathrm{CO}}_{2}\;(g)$$

**Figure 3:**
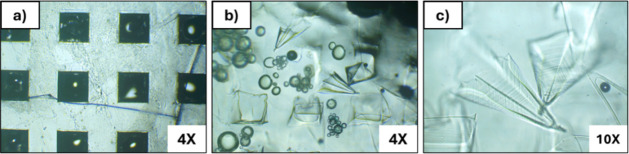
Time-lapse microscopy (top view) of effervescent needle separation: (a) Initial patch configuration (4× magnification); (b) Partial needle detachment after 15 s showing bubble formation (4× magnification); (c) Complete needle separation with needles suspended in surrounding medium after 60 s (10× magnification).

This gas-mediated separation mechanism offers significant advantages over conventional dissolving microneedles by ensuring complete needle deposition in the skin while allowing early patch removal, thereby improving patient comfort and compliance. Notably, the 60-s detachment time observed in our PVA/PVP system is comparable to the rapid separation kinetics reported by Li et al.^11^ for PLGA-based effervescent microneedles, where complete detachment occurred within approximately 10 s in PBS solution. However, the slightly longer detachment time in our system reflects the different polymer dissolution characteristics: while the PVP effervescent backing dissolves rapidly upon contact with aqueous media, the denser PVA microneedle matrix requires additional time for sufficient hydration to initiate separation at the polymer interface. This controlled detachment rate is advantageous for antimicrobial applications, as it ensures adequate time for the microneedles to penetrate through the stratum corneum and reach the viable epidermis before separation occurs, thereby maximizing drug deposition at the target site.

The effervescent approach addresses a critical limitation of traditional DMNs, which typically require prolonged application times (often 10–30 min) for complete needle dissolution, potentially causing patient discomfort and reducing adherence to treatment protocols. In contrast, our system enables patch removal after just 60 s while ensuring that the therapeutic payload remains deposited in the tissue. This rapid detachment mechanism is particularly advantageous for pediatric and geriatric populations, where patient cooperation may be limited, and for applications in resource-limited settings where prolonged monitoring is impractical.

### 3.2 Skin Penetration Performance

The dye-binding study conducted on hairless porcine skin demonstrated successful microneedle penetration with high efficiency. Following removal of excess dye, distinct red spots were observed on the skin surface, confirming that microneedles successfully penetrated the stratum corneum and reached the viable epidermis (Figure 4). Quantitative assessment revealed an average insertion depth of 426.1 ± 16.8 μm, which effectively penetrates through the stratum corneum (approximately 10–20 μm thick) and extends into the epidermal layer (400–1,500 μm thickness depending on anatomical location).

**Figure 4:**
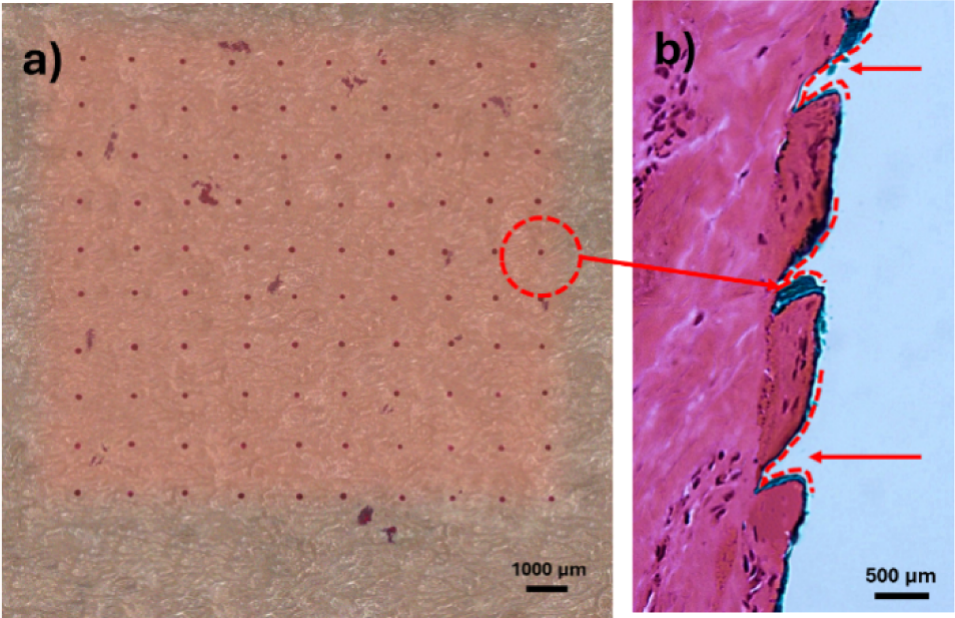
Skin penetration assessment: (a) Top view of insertion sites visualized with tissue-marking dye demonstrating complete TCH-DMNAP array penetration; (b) H&E-stained cross-section showing microneedle insertion depth in porcine dermis.

Penetration efficiency quantification through 10 independent patch applications (*n* = 10) revealed that 92–100 micropores were successfully formed per 100-needle array, corresponding to an average efficiency of 96%. This high penetration rate confirms the mechanical robustness of the fabricated TCH-DMNAPs and validates the thumb-pressure application method for clinical use. Minor variations in penetration efficiency (4% deviation from perfect penetration) can be attributed to natural heterogeneity of the skin surface, including variations in local stiffness, hydration status, and the manual nature of the thumb-pressure application technique. These results are consistent with previous reports on dissolving microneedles, where penetration efficiencies typically range from 85 to 100% depending on needle geometry, material properties, and application method.^32,33^ The 96% penetration efficiency achieved by our PVA-based TCH-DMNAPs compares favorably with the 96 ± 4% detachment efficiency reported for PLGA-based effervescent microneedles by Li et al.,^11^ demonstrating that the water-soluble polymer matrix does not compromise mechanical performance during skin insertion.

The achieved penetration depth of 426.1 ± 16.8 μm is optimal for transdermal drug delivery applications targeting superficial infections. This depth ensures that TCH is delivered directly to the viable epidermis and upper dermis, where bacterial colonization typically occurs in superficial wound infections, while remaining sufficiently shallow to avoid stimulation of dermal nerve endings (located at depths >500 μm), thereby maintaining the painless administration characteristic that is a key advantage of microneedle technology.^34^ Furthermore, this penetration depth bypasses the primary barrier to drug permeation—the stratum corneum—enabling rapid drug absorption and therapeutic action. The consistent penetration depth across replicate applications (coefficient of variation = 3.9%) demonstrates the reproducibility of the fabrication process and the reliability of the mechanical properties.

### 3.3 Mechanical Properties Optimization

Sufficient mechanical strength is essential for DMNAPs to penetrate skin effectively without fracturing or bending during insertion, which could compromise drug delivery efficiency and potentially cause safety concerns.^35^ The required insertion force for successful microneedle penetration typically ranges from 0.1 to 3 N per needle, suitable for manual thumb-pressure application.^34^ Compression testing of various formulations revealed differential mechanical performance based on polymer composition (Figure 5). Mean penetration forces at 0.5 mm displacement were: PVA-0.5/PVP0.75 (0.254 ± 0.043 N/needle), PVA-0.5/PVP1.0 (0.523 ± 0.032 N/needle), PVA-1.0/PVP0.75 (0.848 ± 0.054 N/needle), and PVA-1.0/PVP1.0 (0.492 ± 0.042 N/needle).

**Figure 5:**
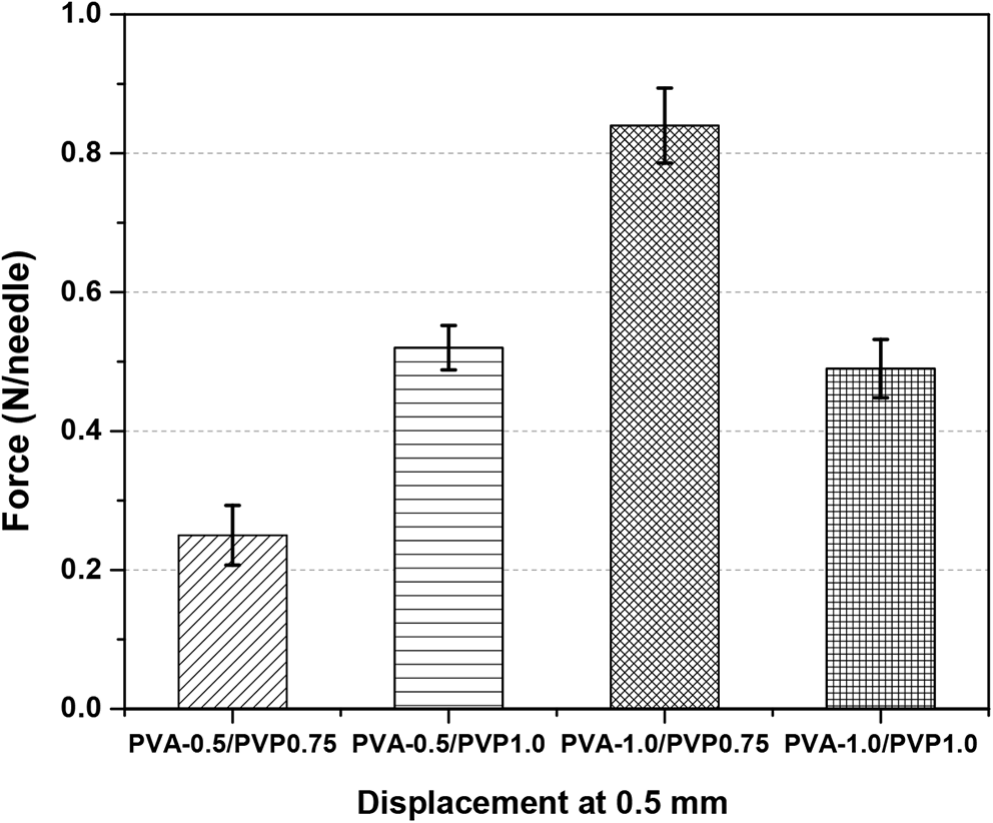
Compression testing of DMNAP formulations at 0.5 mm displacement. Data shown as means ± SD’s (*n* = 3).

The PVA-1.0/PVP0.75 formulation was selected for subsequent TCH loading studies due to its optimal balance of mechanical strength and fabrication consistency. Higher PVA content (1.0 g versus 0.5 g) provides greater structural strength and rigidity, enhancing penetration capability through increased stiffness of the pyramidal needle structures. PVA’s semicrystalline nature and extensive hydrogen bonding networks contribute to this mechanical robustness.^36^ Conversely, PVP’s amorphous nature and plasticizing properties reduce brittleness and function as a stress-absorbing buffer during insertion, helping microneedles maintain their shape without catastrophic fracture.^10^ The intermediate PVP concentration (0.75 g) provides sufficient flexibility to accommodate tissue deformation during insertion while maintaining adequate rigidity for penetration.

The mechanical strength of 0.848 ± 0.054 N/needle achieved by the optimized PVA-1.0/PVP0.75 formulation significantly exceeds the 0.07 N/needle failure force reported for PLGA-based effervescent microneedles.^11^ This substantial difference reflects the distinct mechanical characteristics of the polymer systems: while PLGA microneedles derive their strength from a hydrophobic, slowly biodegrading matrix, PVA microneedles benefit from extensive intermolecular hydrogen bonding that provides high initial stiffness in the dry state. The higher mechanical strength of PVA-based needles offers practical advantages for antimicrobial applications, where consistent penetration through potentially compromised or edematous wound tissue is essential for therapeutic efficacy.

Importantly, TCH incorporation at therapeutic concentrations did not significantly compromise mechanical properties (Figure 6). The insertion forces at 0.5 mm displacement for TCH-DMNAPs remained consistent across drug concentrations: 0.5 TCH-DMNAP (0.892 ± 0.024 N/needle), 1.0 TCH-DMNAP (0.913 ± 0.011 N/needle), and 2.0 TCH-DMNAP (0.936 ± 0.021 N/needle), with no statistically significant differences among formulations (*p* > 0.05). This finding confirms that TCH loading up to 2.0 mg/mL does not interfere with the polymer matrix structure or mechanical integrity, demonstrating excellent compatibility between the drug and carrier materials. The slight trend toward increased insertion force with higher TCH loading (0.892 to 0.936 N/needle) may reflect minor changes in crystallinity or intermolecular interactions within the PVA matrix, though these changes remain within acceptable tolerances for manual application. This mechanical stability across therapeutic dose ranges is crucial for clinical translation, as it ensures consistent performance regardless of the prescribed drug concentration, simplifying manufacturing and quality control processes.

**Figure 6:**
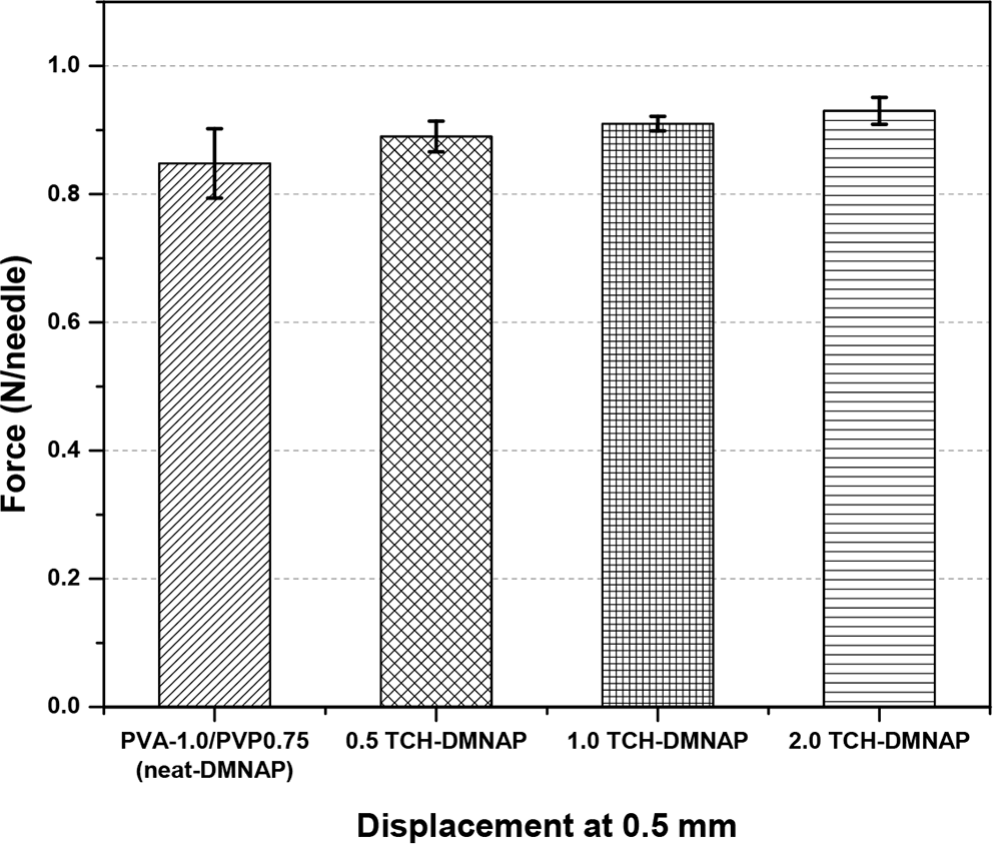
Effect of TCH loading on mechanical properties of neat-DMNAP and TCH-DMNAP samples. *Statistically significant vs neat-DMNAP (*p* < 0.05). Data shown as means ± SD’s (*n* = 3).

### 3.4 Drug Loading and Release Kinetics Analysis

#### 3.4.1 Drug Loading Efficiency

The TCH-DMNAP formulations demonstrated dose-proportional drug loading with excellent reproducibility. Spectrophotometric analysis at λ_max_ = 370 nm revealed drug loadings of 1.14 ± 0.11, 2.23 ± 0.18, and 4.52 ± 0.34 μg/mg for the 0.5, 1.0, and 2.0 TCH-DMNAP formulations, respectively. The strong linear relationship between theoretical and actual drug loading (*R* ^2^ > 0.99) confirmed the reproducibility and scalability of the fabrication process, which is essential for regulatory approval and commercial manufacturing. The relatively low coefficients of variation (9.6%, 8.1%, and 7.5% for the three formulations, respectively) demonstrate the precision of the sequential casting methodology and the uniform distribution of TCH within the PVA matrix. These drug loading values translate to total patch drug contents of approximately 114, 223, and 452 μg per 100-needle array, providing therapeutic flexibility for treating infections of varying severity.

The achieved drug loading efficiency compares favorably with previous reports of TCH-loaded microneedle systems. For example, Zhang et al.^18^ reported TCH loading of 0.5 mg per patch for periodontal applications, while Gao et al.^15^ achieved loading efficiencies of 1–3 mg/mL in hyaluronic acid-based double-layer microneedles for diabetic wounds. Our platform’s ability to accommodate drug concentrations up to 4.52 μg/mg without compromising mechanical integrity or release kinetics demonstrates its versatility for dose optimization based on specific clinical requirements and bacterial resistance profiles.

#### 3.4.2 In Vitro Release Profiles

The cumulative TCH release profiles in PBS (pH 7.4, 37 °C) exhibited characteristic biphasic kinetics with an initial rapid release phase followed by sustained release (Figure 7). Within the first 60 min, 71.84 ± 2.50%, 78.42 ± 1.85%, and 83.39 ± 2.10% of TCH was released from the 0.5, 1.0, and 2.0 TCH-DMNAP formulations, respectively. This initial burst release can be attributed to three synergistic mechanisms: (1) rapid hydration of the hydrophilic PVA matrix upon contact with the aqueous release medium, (2) diffusion of drug molecules present on or near the needle surface where concentration gradients are steepest, and (3) enhanced porosity created by the effervescent CO_2_ generation, which creates microchannels facilitating drug diffusion.^37,38^

**Figure 7:**
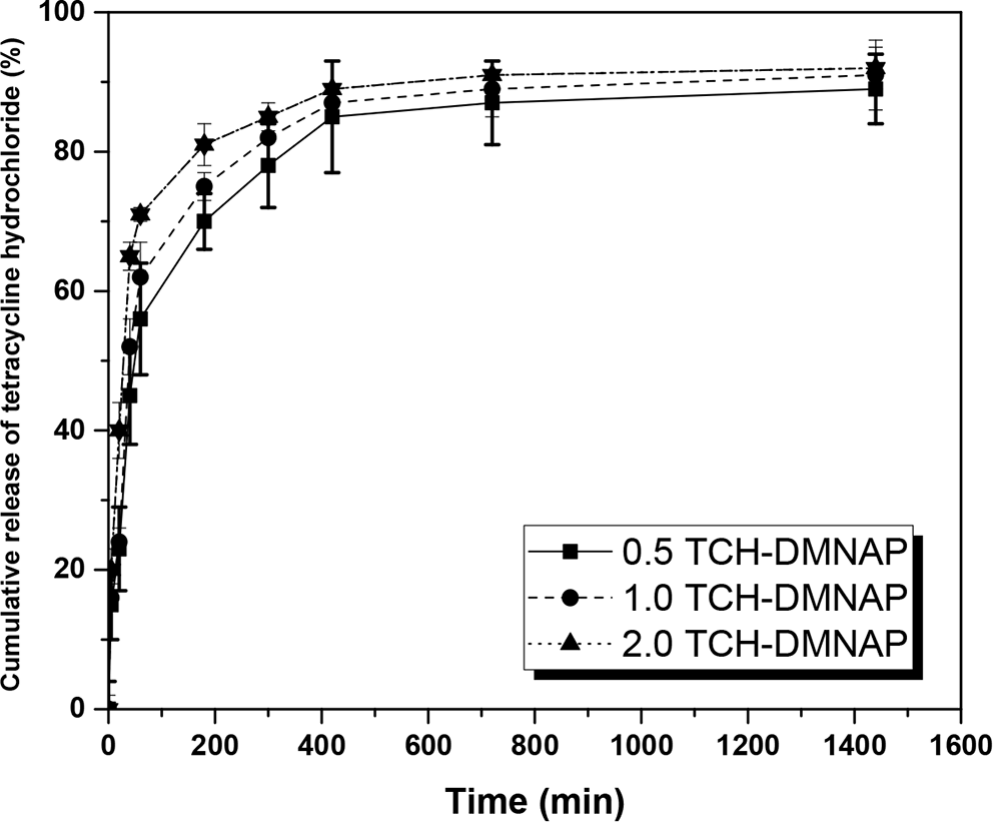
Cumulative TCH release profiles from TCH-DMNAP samples in PBS at 37 °C. Data shown as means ± SD’s (*n* = 3).

The release kinetics of our PVA-based TCH-DMNAPs differ fundamentally from those reported for PLGA-based effervescent microneedles. Li et al.^11^ demonstrated that PLGA microneedles loaded with levonorgestrel exhibited essentially no burst release in PBS, with drug release occurring at a rate of approximately 1.4% per day over at least 60 days—kinetics appropriate for long-acting hormonal contraception but unsuitable for antimicrobial therapy where immediate bactericidal action is required. In contrast, our PVA-based system achieves >70% drug release within the first 60 min, followed by sustained release reaching >90% cumulative delivery within 24 h. This dramatically accelerated release profile is a direct consequence of the hydrophilic, water-soluble nature of PVA compared to the hydrophobic, slowly biodegrading PLGA matrix. The fundamental difference in release mechanism—dissolution-mediated versus degradation-controlled—represents a key innovation enabling translation of effervescent separation technology from long-acting delivery to rapid-onset antimicrobial applications. Similar rapid release profiles have been reported for other hydrophilic microneedle systems: Gao et al.^15^ demonstrated complete TCH release within 25 min from hyaluronic acid-based microneedle tips, and Liu et al.^16^ achieved 91.5% cumulative TCH release within 20 min from their double-layer DMN system. These findings collectively support the suitability of water-soluble polymer matrices for antimicrobial microneedle applications requiring immediate therapeutic action.

According to Figure 7, the subsequent sustained release phase achieved cumulative releases of 88.68 ± 1.45%, 91.34 ± 1.20%, and 92.66 ± 2.15% at 24 h for the respective formulations, demonstrating near-complete drug depletion with minimal residual drug retention in the polymer matrix. The dose-dependent release behavior—where higher drug loading enhances both the rate and extent of release—demonstrates that concentration gradients serve as the primary driving force for diffusion, consistent with Fickian transport mechanisms. This sustained release is clinically advantageous as it maintains therapeutic drug concentrations at the infection site for an extended duration, potentially reducing dosing frequency and improving patient compliance.

The efficient dissolution and consistent release patterns can be attributed to several factors: 1) TCH’s moderate aqueous solubility (∼3.3 mg/mL in PBS at pH 7.2),^39^ which prevents premature precipitation that could impede release; 2) optimal pH conditions (7.4) that maintain TCH in its ionized, water-soluble form; 3) the hydrophilic nature of the PVA carrier matrix, which facilitates water penetration and polymer chain relaxation;^37,38^ and 4) the limited crystallinity of the PVA matrix due to ambient temperature drying, which creates a more amorphous structure conducive to rapid dissolution.^36^ The biphasic release profile is ideally suited for wound infection treatment, where rapid initial release addresses acute bacterial colonization, while sustained release provides prolonged antimicrobial coverage during the critical early healing phase.

#### 3.4.3 Release Kinetics Modeling and Mechanism Analysis

Mathematical modeling of the release data provides mechanistic insights into the drug transport processes governing TCH delivery from the DMNAPs (Table 2). The Korsmeyer–Peppas model provided the best fit across all formulations, with *R* ^2^ values exceeding 0.93 and release exponent (*n*) values ranging from 0.43 to 0.45. These *n* values indicate quasi-Fickian diffusion, suggesting that drug release is predominantly governed by diffusion with minor contributions from polymer matrix relaxation.^40^ This release mechanism is consistent with previous reports on PVA-based dissolving microneedles and reflects the relatively rapid hydration and dissolution characteristics of the polymer matrix.

**Table 2:** Mathematical Modeling of TCH Release Kinetics

	Korsmeyer–Peppas			Higuchi		Zero-order		First-order	
Samples	*K*_KP_ (min^–*n*^)	*R* ^2^	n	*K*_H_ (min^–0.5^)	*R* ^2^	*K*_1_ (min^–1^)	*R* ^2^	*K*_0_ (min^–1^)	*R* ^2^
0.5 TCH-DMNAP	28.42	0.936	0.43	3.84	0.812	0.062	0.524	0.002	0.487
1 TCH-DMNAP	31.85	0.941	0.44	3.92	0.825	0.064	0.538	0.002	0.502
2 TCH-DMNAP	35.29	0.947	0.45	4.01	0.837	0.065	0.551	0.002	0.516

The Higuchi model, which describes drug release from matrix systems where diffusion is the rate-limiting step, showed moderate correlation (*R* ^2^ = 0.812–0.837), further supporting the diffusion-controlled release mechanism.^41^ The Higuchi rate constants (*K*_H_) increased with drug loading (3.84 to 4.01 min^–0.5^), reflecting enhanced diffusional driving forces at higher drug concentrations. The relatively poor fits obtained with zero-order (*R* ^2^ = 0.524–0.551) and first-order models (*R* ^2^ = 0.487–0.516) indicate that drug release is not solely dependent on constant surface erosion (zero-order) or simple concentration-dependent diffusion from a uniform matrix (first-order).

The biphasic release profile can be mechanistically explained by the structural evolution of the hydrating PVA matrix. During the initial rapid release phase (0–60 min), water rapidly penetrates the outer layers of the microneedles, causing immediate swelling and dissolution of the most accessible polymer chains. Drug molecules located near the surface or within loosely packed polymer regions diffuse rapidly along the steep concentration gradient into the surrounding medium. This phase is further accelerated by the effervescent separation mechanism, which generates CO_2_ bubbles that create transient microchannels and increase the effective surface area for drug release.

In the subsequent sustained release phase (60–1440 min), drug diffusion becomes increasingly controlled by the tortuous pathways through the swelling polymer network. As the PVA matrix undergoes progressive dissolution, the diffusion path length increases and polymer chain entanglements create temporary barriers to drug transport, resulting in slower release rates. The decreasing concentration gradient as drug is depleted from the matrix also contributes to the deceleration of release. This sustained phase maintains therapeutic drug levels while avoiding potential toxicity from excessive burst release.

The dose-dependent increase in release rate constants (*K*_KP_ values from 28.42 to 35.29 min–^*n*^) observed across formulations suggests that higher drug loading creates steeper concentration gradients, thereby enhancing the diffusional driving force. This finding has important implications for clinical translation, as it allows for tunable release kinetics through simple adjustment of initial drug loading. Importantly, all formulations maintained drug concentrations well above the established MIC values (0.15 μg/mL for *E. coli* and 0.04 μg/mL for *S. aureus*) throughout the 24-h study period, ensuring sustained antimicrobial efficacy. Even the lowest drug loading formulation (0.5 TCH-DMNAP) would theoretically maintain concentrations exceeding 10 × MIC for at least 24 h, providing a substantial safety margin against subtherapeutic drug levels.

The consistent Korsmeyer–Peppas model fit across different drug loadings, combined with the predictable dose-dependent release behavior, confirms the robustness of this platform for controlled antimicrobial delivery. The quasi-Fickian diffusion mechanism ensures that release kinetics are governed by well-understood physical principles, facilitating quality-by-design approaches for manufacturing scale-up and regulatory submission. Furthermore, the rapid initial release combined with sustained delivery addresses both the immediate need for bacterial suppression and the ongoing requirement for infection control during the critical early wound healing phase.

### 3.5 Antimicrobial Efficacy

#### 3.5.1 MIC and MBC Determination

To establish rational drug loading targets for the microneedle formulations, MIC and MBC values were first determined for TCH against the test strains (Table 3). TCH demonstrated differential activity against Gram-positive and Gram-negative bacteria, consistent with its mechanism targeting bacterial ribosomes. *S. aureus* ATCC 25923 showed greater sensitivity with MIC and MBC values of 0.04 and 2.44 μg/mL, respectively, while *E. coli* ATCC 25922 exhibited higher tolerance with MIC and MBC values of 0.15 and 9.77 μg/mL, respectively. The differential susceptibility of *E. coli* (Gram-negative) versus *S. aureus* (Gram-positive) reflects fundamental differences in bacterial cell wall architecture. Gram-negative bacteria possess an outer membrane containing lipopolysaccharides that serves as an additional permeability barrier, reducing antibiotic penetration and increasing resistance.^13^ In contrast, Gram-positive bacteria lack this outer membrane, rendering them generally more susceptible to antibiotics like tetracycline.

**Table 3:** MIC and MBC Values of TCH Tested by the Microdilution Technique

Test microorganism	Gram type	MIC^a^ (μg/mL)	MBC^b^ (μg/mL)
*E. coli* ATCC 25922	–	0.15	9.77
*S. aureus* ATCC 25923	+	0.04	2.44

a MIC is the minimum inhibitory concentration and.

b MBC is the minimum bactericidal concentration.

To ensure robust antibacterial suppression in wound applications where biofilm formation and tissue debris may impair drug efficacy, TCH concentrations were deliberately selected at four times the MIC values (4 × MIC: approximately 0.61 μg/mL for *E. coli* and 0.15 μg/mL for *S. aureus*). Consequently, the formulated TCH concentrations of 0.5, 1.0, and 2.0 mg/mL in the casting solutions greatly exceed these thresholds, providing substantial safety margins against subtherapeutic dosing and potential resistance development. This conservative dosing strategy is particularly important for treating infections in compromised tissue environments, such as chronic wounds or diabetic ulcers, where impaired vascularity and host defenses necessitate higher local antibiotic concentrations to achieve bacterial eradication.

#### 3.5.2 Time-Kill Kinetics

Time-kill assays provide dynamic assessment of bactericidal activity, revealing the rate and extent of bacterial reduction over time—information critical for understanding therapeutic effectiveness. Against *E. coli* ATCC 25922 (Figure 8a), control and neat-DMNAP samples exhibited modest initial growth during the first hour, followed by exponential increase and stabilization after 3 h, reaching plateau densities of approximately 5 × 10^6^ CFU/mL. The neat-DMNAP sample mirrored the control behavior, confirming the absence of inherent antimicrobial activity in the polymer matrix components (PVA and PVP).

**Figure 8:**
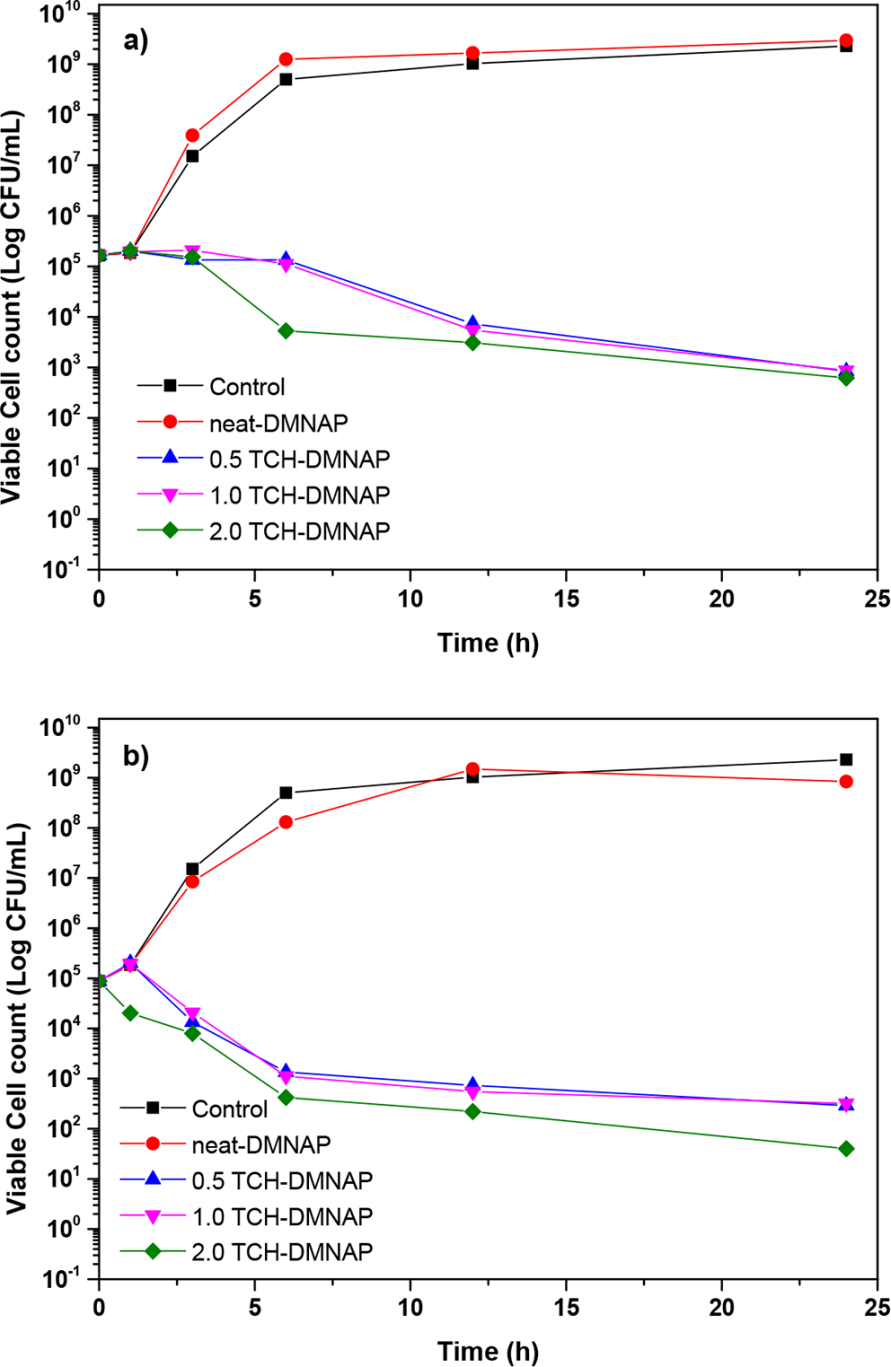
Time-kill kinetics against (a) *E. coli* ATCC 25922 and (b) *S. aureus* ATCC 25923. Data shown as means ± SD’s (*n* = 3).

In stark contrast, all TCH-DMNAP formulations demonstrated potent bactericidal activity. Bacterial counts initially increased to 1.85 × 10^5^ CFU/mL after 1 h, likely reflecting residual viable bacteria from the inoculum, before declining dramatically by 3 h as TCH release reached therapeutic levels. The highest loading formulation (2.0 TCH-DMNAP) exhibited superior performance, achieving bacterial reduction to 5.30 × 10^3^ CFU/mL at 6 h (99.99% reduction relative to control), and further decreasing to 3.10 × 10^3^ and 6.20 × 10^2^ CFU/mL at 12 and 24 h, respectively, maintaining >99.99% reduction throughout the study period.

Against *S. aureus* ATCC 25923 (Figure 8b), reduction patterns paralleled those observed with *E. coli*, reflecting TCH’s broad-spectrum activity. After 24 h, bacterial counts were reduced to 2.90 × 10^2^, 3.20 × 10^2^, and 4.00 × 10^1^ CFU/mL for 0.5, 1.0, and 2.0 TCH-DMNAP formulations, respectively, all achieving >99.99% bacterial elimination. This potent antimicrobial effect stems from TCH’s well-characterized mechanism of binding to the 30S ribosomal subunit, specifically the 16S rRNA, which prevents aminoacyl-tRNA attachment to the ribosomal A site.^13^ This interference with protein synthesis halts bacterial growth and, at higher concentrations, leads to bactericidal effects through accumulation of incomplete proteins and metabolic dysfunction.

The therapeutic efficacy observed in time-kill assays directly correlates with the predetermined MIC/MBC values and release kinetics. Each microneedle patch was designed to deliver TCH at concentrations exceeding 4 × MIC, ensuring bactericidal activity while minimizing the risk of sublethal antibiotic exposure that could promote resistance development. The 2.0 TCH-DMNAP formulation achieved >92% cumulative drug release within 24 h (Figure 7), maintaining sustained therapeutic concentrations that correlated directly with the observed >99.99% bacterial reduction in time-kill studies. The dose-dependent enhancement in bactericidal activity (0.5 < 1.0 < 2.0 mg/mL formulations) confirms that higher drug loading provides more rapid and complete bacterial eradication, which may be particularly advantageous for treating aggressive or antibiotic-resistant infections.

The rapid bactericidal action observed within 6 h is clinically significant, as early intervention in wound infections dramatically improves healing outcomes and reduces the risk of systemic complications such as sepsis. The sustained antimicrobial activity maintained throughout 24 h ensures continuous bacterial suppression during the critical early post-application period, potentially preventing recolonization and biofilm reformation. These results suggest that a single daily application of TCH-DMNAP could provide adequate infection control for superficial wound infections, improving patient convenience and compliance compared to multiple daily applications of topical creams or systemic antibiotic regimens.

#### 3.5.3 Preliminary Qualitative Assessment of Antimicrobial Activity Using an Infected Porcine Skin Model

To bridge the gap between controlled in vitro time-kill assays and future in vivo efficacy studies, we conducted qualitative ex vivo antimicrobial assessment using infected porcine skin as a clinically relevant tissue model. This approach simulates the complex tissue environment encountered in real wound infections, including three-dimensional tissue architecture, extracellular matrix components, and tissue fluid dynamics that may influence drug distribution and bacterial survival.

The qualitative results (Figure 9) demonstrated striking differences in antimicrobial efficacy across treatment groups. For *E. coli* infections, both the untreated infected control and neat-DMNAP samples exhibited dense and uniform bacterial growth across the agar surface, confirming robust colonization of the skin and the absence of any intrinsic antimicrobial effect from the polymeric microneedle matrix. In stark contrast, the agar plate obtained from the 2.0 TCH-DMNAP-treated skin showed an almost complete absence of visible colonies, demonstrating an absence of visible colony growth and indicating near-complete suppression of Gram-negative bacteria following 24 h of localized TCH delivery. For *S. aureus* infections, a similar qualitative trend was observed, although the response was comparatively less pronounced. Both the untreated and neat-DMNAP groups produced confluent bacterial growth, whereas the 2.0 TCH-DMNAP treatment resulted in noticeably reduced colony density, indicating partial but substantial suppression of Gram-positive bacterial survival. The presence of residual *S. aureus* colonies in the TCH-treated group cannot be attributed to intrinsic antibiotic susceptibility, as *S. aureus* exhibits a lower planktonic MBC than *E. coli* (Table 3), and instead likely reflects local tissue-associated protection mechanisms—such as strong surface adhesion, early biofilm formation, protein binding, or spatial heterogeneity in drug distribution—that limit antibiotic access in complex tissue environments.

**Figure 9:**
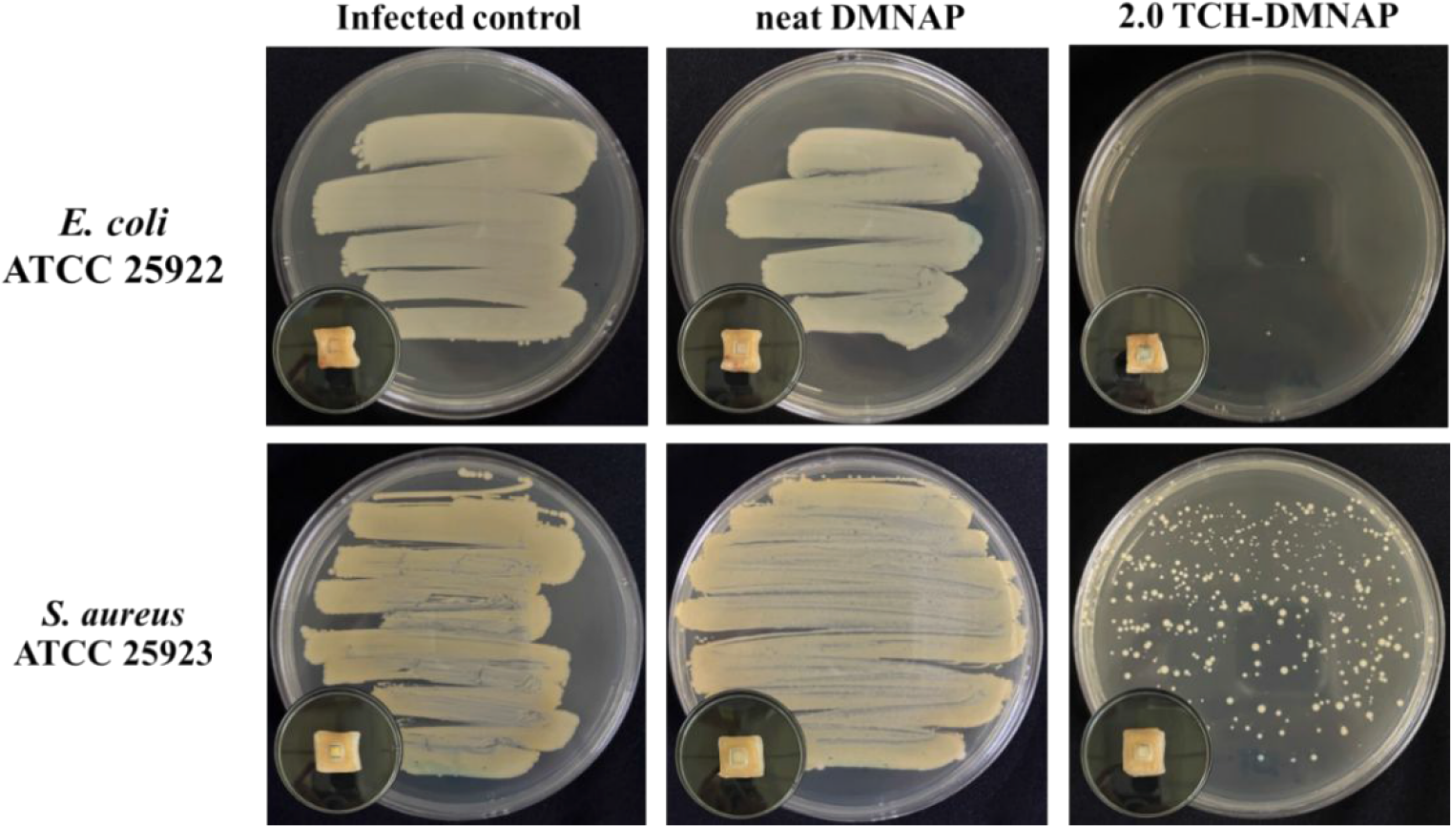
Swab-streak agar results from infected porcine skin treated with 2.0 TCH-DMNAP, neat DMNAP, or untreated control, with inset images showing the microneedle application site prior to sampling.

The differential efficacy against *E. coli* (complete eradication) versus *S. aureus* (substantial but incomplete suppression) warrants careful consideration in the context of the qualitative swab-streak methodology employed. While this technique provides rapid visual assessment of antimicrobial activity suitable for preliminary screening, it has inherent limitations that must be acknowledged. The swab-streak method samples only surface-accessible bacteria and may underestimate total bacterial burden, particularly for *S. aureus*, which exhibits strong adherence to tissue surfaces through surface adhesins and protein A-mediated binding to extracellular matrix components.^32^ Additionally, Gram-positive bacteria like *S. aureus* typically demonstrate greater capacity for biofilm formation and deeper tissue penetration compared to Gram-negative organisms, potentially creating microenvironments with reduced antibiotic access that would not be fully captured by surface swabbing.

Several factors beyond intrinsic bacterial susceptibility may contribute to the observed differential response. First, the inoculum effect—where higher bacterial densities reduce antibiotic efficacy—may play a role if *S. aureus* colonization density exceeded that of *E. coli* at the time of treatment due to differences in growth kinetics or tissue adhesion efficiency. Second, the hydrophilic nature of TCH may favor distribution in aqueous tissue compartments over lipophilic bacterial cell membranes or the lipid-rich biofilm matrices that *S. aureus* commonly produces, potentially limiting access to adherent Gram-positive bacteria. Third, *S. aureus* possesses multiple efflux pumps and can acquire tetracycline resistance determinants, although the ATCC 25923 strain used is documented as tetracycline-susceptible. Similar differential responses between Gram-positive and Gram-negative bacteria have been reported in other TCH-loaded microneedle systems (e.g., Gao et al.^15^ reporting that inhibition zones for *E. coli* (13.1–20.8 mm) were consistently smaller than those for *S. aureus* (21.9–27.5 mm) in agar diffusion assays); however, agar diffusion assays and ex vivo tissue models are not directly comparable due to fundamental differences in drug transport, tissue binding, and bacterial spatial distribution.

We acknowledge that the qualitative swab-streak methodology employed in this study, while appropriate for preliminary assessment, provides limited quantitative resolution and cannot determine absolute bacterial burden within the tissue. Quantitative tissue homogenization with serial dilution and colony counting would provide more rigorous determination of log reduction values and enable statistical comparison across treatment groups. However, the dramatic visual difference between TCH-treated and control samples—particularly the complete absence of *E. coli* colonies versus confluent growth in controls—provides compelling preliminary evidence of antimicrobial efficacy in a complex tissue environment. The marked reduction in *S. aureus* colony density, while incomplete, nonetheless demonstrates clinically meaningful antimicrobial activity against Gram-positive pathogens. Future studies employing quantitative tissue homogenization, confocal microscopy to visualize bacterial distribution within tissue layers, and extended time-course sampling will be essential to fully characterize the antimicrobial performance of this platform and are currently in preparation.

Nevertheless, the marked reduction in *S. aureus* colony density relative to both control groups confirms that the 2.0 TCH-DMNAP achieves clinically meaningful antimicrobial activity against Gram-positive pathogens, even if complete sterilization was not achieved under these ex vivo conditions. In clinical wound infections, the combination of antibiotic therapy with host immune responses (absent in our ex vivo model) would likely synergize to achieve complete bacterial clearance. Collectively, these ex vivo results provide preliminary evidence supporting the translational potential of the effervescent dissolving microneedle platform for topical antimicrobial therapy. The ability to nearly eliminate *E. coli* and substantially suppress *S. aureus* in an infected tissue environment—despite the complex barriers to drug delivery presented by tissue architecture and bacterial colonization—strongly supports progression to in vivo efficacy studies in animal wound models. Future investigations should include quantitative bacterial burden measurements (via tissue homogenization and culture), histological assessment of tissue inflammation and healing, and evaluation of formulations with even higher TCH loading or combination antibiotics to achieve complete eradication of resistant Gram-positive pathogens.

### 3.6 Biocompatibility Assessment

The in vitro cytotoxicity of TCH-DMNAP formulations was evaluated using HDFs through the indirect MTT assay at 24 h (Figure 10), providing essential safety data to guide clinical dose selection. According to ISO 10993–5:2009 standards for biological evaluation of medical devices, cell viability ≥70% is considered noncytotoxic and acceptable for biomedical applications.^27^ Our results demonstrated a dose-dependent relationship between TCH concentration and cell viability: neat-DMNAP (91.25%), 0.5 TCH-DMNAP (87.65%), 1.0 TCH-DMNAP (82.66%), and 2.0 TCH-DMNAP (60.34%).

**Figure 10:**
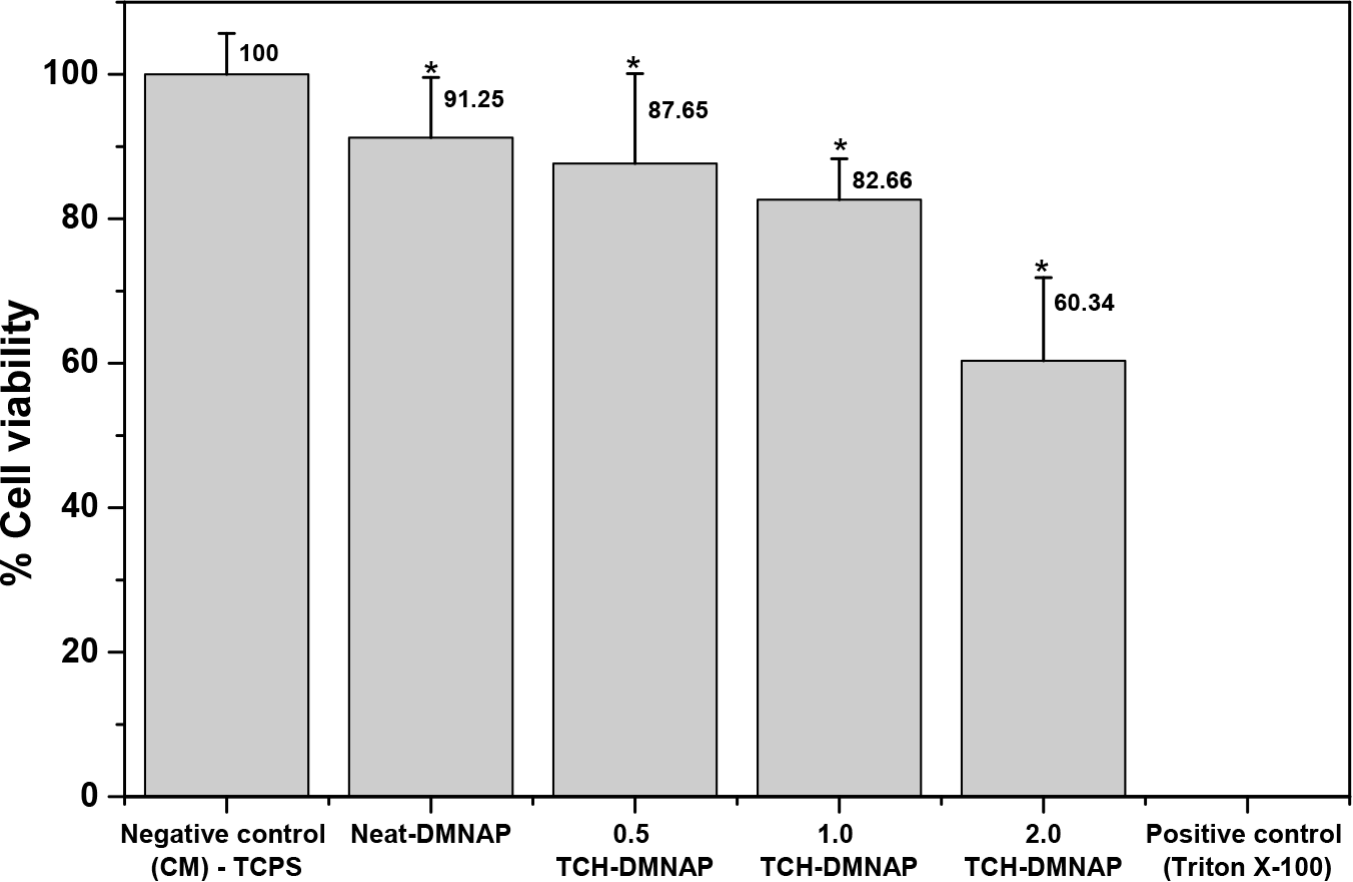
HDF viability assessment via MTT assay, relative to negative control cells (CM, 100%), after exposure to neat DMNAP, 0.5, 1.0, 2.0 TCH-DMNAP and Triton X-100 as positive control. *Statistically significant vs control (*p* < 0.05). Data shown as means ± SD’s (*n* = 3).

The neat-DMNAP exhibited excellent biocompatibility (91.25% viability), confirming that the PVA and PVP polymer matrix components are nontoxic and suitable for topical application, consistent with their established use in FDA-approved medical devices and pharmaceutical formulations. TCH-loaded formulations at 0.5 and 1.0 mg/mL maintained viabilities of 87.65% and 82.66%, respectively—both exceeding the 70% safety threshold by comfortable margins. These formulations thus represent the optimal therapeutic window, providing potent antimicrobial efficacy (>99.99% bacterial reduction; Figure 8) while maintaining acceptable biocompatibility. The 2.0 TCH-DMNAP formulation exhibited reduced viability (60.34%), falling slightly below the ISO 10993–5 threshold. This finding suggests that 2.0 mg/mL represents the upper limit of safe TCH loading for this platform. The dose-dependent cytotoxicity likely reflects TCH’s known off-target effects at elevated concentrations, including mitochondrial dysfunction through inhibition of mammalian mitochondrial ribosomes (which share structural similarities with bacterial ribosomes), generation of reactive oxygen species, and potential interference with collagen synthesis in fibroblasts.^19,42^

However, it is important to contextualize this finding within the broader therapeutic landscape. First, the indirect MTT assay represents a conservative worst-case scenario, as cells are continuously exposed to extract medium containing leached materials for 24 h. In actual clinical use, drug release is transient and diluted by tissue fluid dynamics, substantially reducing cellular exposure. Second, the localized nature of microneedle delivery confines drug exposure primarily to the epidermal layer, minimizing systemic absorption and reducing risks to internal organs. Third, mild transient cytotoxicity may be acceptable in the context of treating life-threatening wound infections, where the benefits of rapid bacterial eradication outweigh minor localized tissue irritation that typically resolves during the normal wound healing process.

Previous studies on tetracycline biocompatibility in various formulations have reported similar dose-dependent cytotoxicity patterns, with therapeutic concentrations generally exhibiting minimal toxicity to mammalian cells while maintaining potent antibacterial activity.^23^ For instance, Dirain and Antonelli^42^ reported that tetracycline concentrations below 100 μg/mL maintained >80% viability in human tympanic membrane fibroblasts, while higher concentrations induced mitochondrial damage and apoptosis. Our findings are consistent with this literature, confirming that careful dose optimization is essential for balancing antimicrobial efficacy with tissue safety.

Based on these comprehensive biocompatibility data, we recommend the 1.0 TCH-DMNAP formulation as the lead candidate for in vivo efficacy and safety studies. This formulation achieved 82.66% cell viability (exceeding the ISO safety threshold), demonstrated >99.99% bacterial reduction in time-kill assays (Figure 8), and provided sustained drug release for 24 h (Figure 7), representing an optimal balance of efficacy, safety, and manufacturability. For clinical translation, regulatory submissions should include additional safety assessments such as skin irritation testing (ISO 10993–10), sensitization studies, and acute/subchronic toxicity evaluations to fully characterize the risk-benefit profile.

## 4 Conclusion

This study successfully developed and characterized a novel effervescent-assisted dissolving microneedle array patch system for enhanced transdermal delivery of tetracycline hydrochloride. The innovative three-layer architecture—comprising PVA-based drug-loaded microneedles, an effervescent separation layer utilizing sodium bicarbonate/tartaric acid chemistry, and a PVP backing for structural support—achieved synergistic integration of mechanical robustness, controlled drug release, and rapid needle detachment capabilities. This platform extends the effervescent separation concept pioneered by Li et al.^11^ for long-acting hormonal delivery to the distinct therapeutic context of rapid-onset antimicrobial therapy, demonstrating that deliberate selection of hydrophilic polymer matrices (PVA/PVP) enables biphasic release kinetics ideally suited for wound infection treatment. This platform addresses several critical limitations of existing microneedle technologies while maintaining the core advantages of painless administration and potential for self-application.

The optimized PVA-1.0/PVP0.75 formulation demonstrated sufficient mechanical strength (0.848 ± 0.054 N/needle) for reliable skin penetration, achieving insertion depths of 426.1 ± 16.8 μm with 96% penetration efficiency in ex vivo porcine skin. These mechanical properties remained consistent across drug loading ranges, confirming the compatibility of TCH with the polymer matrix. The effervescent separation mechanism enabled complete needle detachment within 60 s through CO_2_-mediated layer separation, representing a significant advance over conventional dissolving microneedles that require prolonged application times (typically 10–30 min). This rapid detachment feature directly addresses patient comfort concerns and facilitates clinical adoption, particularly in pediatric and geriatric populations where extended application times may be impractical.

The drug release profile exhibited ideal biphasic kinetics, combining rapid initial burst release (>70% within 60 min) for immediate antimicrobial action with sustained release achieving >90% cumulative delivery within 24 h. Mathematical modeling confirmed that release follows quasi-Fickian diffusion kinetics (Korsmeyer–Peppas model: *R* ^2^ > 0.93, *n* = 0.43–0.45), providing mechanistic understanding essential for quality-by-design manufacturing approaches. The dose-dependent release behavior allows for tunable therapeutic profiles through simple adjustment of initial drug loading, offering flexibility for treating infections of varying severity.

Comprehensive antimicrobial evaluation demonstrated potent bactericidal efficacy against both Gram-positive (*S. aureus* ATCC 25923) and Gram-negative (*E. coli* ATCC 25922) bacteria. Time-kill kinetics revealed >99.99% bacterial reduction within 6 h at concentrations exceeding 4 × MIC, with sustained activity maintained throughout 24 h. Preliminary qualitative ex vivo assessment using infected porcine skin confirmed near-complete elimination of *E. coli* and substantial suppression of *S. aureus*, providing preliminary validation of the platform’s efficacy in complex tissue environments. While the qualitative swab-streak methodology employed has inherent limitations for precise bacterial enumeration, the dramatic visual differences between treated and control samples strongly support the antimicrobial efficacy of TCH-DMNAPs. Quantitative tissue homogenization studies are warranted to fully characterize bacterial log reduction values and are planned for subsequent investigations. These results strongly support progression to in vivo efficacy studies in animal wound models.

Biocompatibility assessment using human dermal fibroblasts confirmed acceptable cell viability (>82%) for therapeutic formulations containing 0.5–1.0 mg/mL TCH, meeting ISO 10993–5 standards for medical device safety. The dose-dependent cytotoxicity profile identified 1.0 mg/mL TCH as the optimal therapeutic concentration, balancing potent antimicrobial activity with tissue safety. The 2.0 mg/mL formulation, while demonstrating superior bactericidal efficacy, exhibited reduced cell viability (60.34%), suggesting this concentration represents the upper safety limit requiring careful risk-benefit evaluation for clinical applications.

The effervescent-assisted DMNAP platform offers several translational advantages: 1) painless, minimally invasive administration enhancing patient acceptance and compliance; 2) localized high-dose antibiotic delivery achieving tissue concentrations that may overcome resistance mechanisms while minimizing systemic exposure and side effects; 3) rapid needle detachment enabling convenient self-application without prolonged patch retention; 4) sustained drug release providing 24-h antimicrobial coverage with once-daily application; 5) elimination of sharps waste and needle-stick injury risks; and 6) scalable manufacturing using simple sequential casting methodology compatible with Good Manufacturing Practice (GMP) requirements.
